# sGCα_1_-deficient mice: a novel murine model of spontaneous primary open angle glaucoma

**DOI:** 10.1186/1471-2210-11-S1-P13

**Published:** 2011-08-01

**Authors:** Emmanuel S Buys, Yu-Chieh Ko, Sarah Hayton, Alexander Jones, Laurel Tainsh, Ruiyi Ren, Andrea Giani, Emma Abernathy, Haiyan Gong, Douglas J Rhee, Peter Brouckaert, Meredith Gregory, Louis R Pasquale, Kenneth D Bloch, Bruce Ksander

**Affiliations:** 1 Department of Anesthesia, Critical Care and Pain Medicine, Massachusetts General Hospital, Boston, MA, USA; 2 Department of Ophthalmology, Schepens Eye Research Institute, Boston, MA, USA; 3 Department of Ophthalmology, Boston University School of Medicine, Boston, MA, USA; 4 Department of Ophthalmology, Massachusetts Eye and Ear Infirmary, Boston, MA, USA; 5 VIB Department of Molecular Biomedical Research, Ghent University, Ghent, Belgium

## Background

Primary open angle glaucoma (POAG) is a progressive eye disease that leads to blindness due to the irreversible loss of retinal ganglion cells and degeneration of the optic nerve (ON). As of yet, there is no cure for glaucoma. Although available therapies delay disease progression, they offer incomplete protection. Elevated intraocular pressure (IOP) is an important risk factor for POAG. However, the exact molecular mechanisms that trigger increased IOP and glaucomatous optic neuropathy remain incompletely understood. While a few spontaneous murine models of glaucoma exist, none are models of POAG, the most prevalent form of glaucoma. Impaired nitric oxide (NO) signaling has been implicated in the development of glaucoma. To further test the hypothesis that impaired NO/cGMP signaling contributes to the pathogenesis of POAG, we tested whether mice lacking the α_1_ subunit of the NO receptor soluble guanylate cyclase (sGCα_1_^-/-^ mice) develop POAG.

## Materials and methods

IOP was measured serially using a TonoLab-Tonometer in age-matched female wild-type (WT) and sGCα_1_^-/-^ mice. Iridocorneal angle and trabecular meshwork in WT and sGCα1^-/-^ mice were examined by light and transmission electron microscopy. Aqueous humor turnover was assessed non-invasively using a fluorophotometric approach. Retinal nerve fiber layer (RNFL) thickness was measured via optical coherence tomography (SD-OCT, Bioptigen). Axons in ON cross-sections, stained with paraphenylenediamine, were counted.

## Results

IOP increased significantly with age in sGCα_1_^-/-^ but not in WT mice. Morphological analysis revealed an open iridocorneal angle in eyes from sGCα_1_^-/-^ mice with high IOP. Aqueous humor turnover rates were lower in sGCα_1_^-/-^ than in WT mice, suggesting that increased outflow resistance underlies the elevated IOP. An age-related thinning of the RNFL, consistent with glaucomatous injury, was observed in sGCα_1_^-/-^ but not in WT mice. Similarly, axon counts were lower in ON isolated from sGCα_1_^-/-^ than from WT mice.

## Conclusion

sGCα_1_-deficiency is associated with increased IOP, decreased aqueous humor outflow, thinning of the RNFL and decreased axon counts in the ON. These findings support our hypothesis that sGCα_1_^-/-^ mice develop POAG. Identifying a well-characterized signaling pathway (NO/cGMP signaling) as an important regulatory mechanism controlling outflow and IOP is expected to contribute significantly to our understanding of POAG pathogenesis and may inform the clinical development of existing cGMP-elevating therapeutic compounds for the treatment of elevated IOP and POAG. Moreover, the sGCα_1_^-/-^ mouse model for POAG provides a novel and important tool to test other strategies for disease prevention and treatment of high IOP and POAG.

